# An Isolated Plantar Medial Navicular Dislocation Without Associated Body Fracture Following Low Energy Trauma

**DOI:** 10.1155/cro/9994767

**Published:** 2026-04-30

**Authors:** Saad Mohammad, Roy Small, Matthew Erbst, Christopher Nicholas

**Affiliations:** ^1^ Department of Orthopedic Surgery, Michigan State University–McLaren Macomb Medical Center, Clinton Township, Micihigan, USA; ^2^ Department of Orthopedic Surgery, McLaren Macomb–Center for Orthopedic Surgery, Clinton Township, Micihigan, USA

## Abstract

The occurrence of isolated tarsal navicular dislocation without fracture is infrequently documented in the orthopedic literature. Over the past century, there have been approximately 15 cases reported of isolated navicular dislocation without a fracture. The majority of these cases resulted from high‐energy mechanisms with limited follow‐up. The injury mechanism has commonly been explained as a pronation–abduction force applied to the midfoot, leading to a medial dislocation of the navicular with disruption of its ligamentous support. We present a unique case of a 57‐year‐old female who sustained an isolated tarsal navicular dislocation without fracture following a low‐energy mechanism. She underwent successful closed reduction, followed by Kirschner wire fixation of the midfoot in the operating room. The patient was compliant with postoperative recommendations and regularly attended follow‐up appointments. Significant progress was noted in pain relief and functional improvement at her 8‐month follow‐up. Nonetheless, certain long‐term sequelae persisted, manifesting as mild chronic midfoot pain by her 15‐month follow up. The low energy mechanism in this case not only deviates from what has been previously documented in the literature regarding isolated navicular dislocations but also includes thorough follow‐up, a feature often lacking in similar cases.

## 1. Introduction

Complete dislocation of the tarsal navicular bone without a body fracture is an extremely rare injury [1]. The literature pertaining to isolated dislocation of the navicular from its articulations with the talus, cuboid, and cuneiforms is scarce. The literature that does exist regarding complete dislocation of the navicular is associated with high‐energy injuries [2–5], fracture [6], or is accompanied by other midfoot ligamentous injuries [1]. The tarsal navicular bone is the keystone of the medial longitudinal arch and is stabilized by rigid dorsal and plantar ligaments, rendering it more prone to fracture than dislocation [3, 7]. The navicular bears the majority of the load applied to the foot within the tarsal complex during weight bearing [8]. Previous literature suggests that isolated navicular dislocation without a fracture is anatomically impossible [6, 9] and that the medial and lateral longitudinal columns of the foot must be disrupted in order for the navicular to completely dislocate [1, 3]. When discussing the classification of a truly “isolated” navicular dislocation, the definition of such is a subject of technical debate. Although Pathria et al. [10] describes it as rare phenomena, when it is mentioned in orthopedic literature it is frequently used to describe an absence of fracture of the navicular body itself, not necessarily as absence of midfoot injury. This comes with the assumption that there is ligamentous injury to the capsuloligamentous complex of the navicular. In other literature, especially that of sports medicine, the notation of pure joint dislocation as described in Jung et al. [11] implies the lack of further midfoot injury. This is in opposition to other literature that states that there must be further significant osseous and ligamentous failure of the midfoot. [1, 3] In 1999, Dhillon and Nagi published a case series on six patients with dislocation of the tarsal navicular and all were secondary to high‐energy mechanisms with further associated injury substantial midfoot injury. Our literature review demonstrated there were four reported cases published in the last 10 years regarding isolated complete navicular dislocation without navicular fracture [2–5]. All were secondary to high‐energy injuries. In 2013, Davis et al. reported a total of only 15 cases dating back to 1924 [3]. Multiple treatment options have been described including closed reduction and casting, closed reduction and percutaneous pinning (CRPP), open reduction internal fixation, and open reduction with primary arthrodesis ([2–7, 9, 12]; “Total Dislocation of the Navicular,” 1999). To our knowledge, there have been no reported cases of isolated navicular dislocation without associated body fracture or concurrent significant midfoot ligamentous injury following a low‐energy trauma. In this case report, we demonstrate an isolated plantar medial navicular dislocation without associated body fracture in a patient involved in a low‐energy trauma. This is with notation that within our fracture pattern, there was osseous cuboid and calcaneal injury, so as to prior discussion, this would refer to the former definition of isolated as compared with the latter, specifically referring to lack of navicular body fracture, but still presence of hindfoot and midfoot trauma, ours being unique in the lack of significant midfoot ligamentous instability.

## 2. Case Report

**Figure 1:**
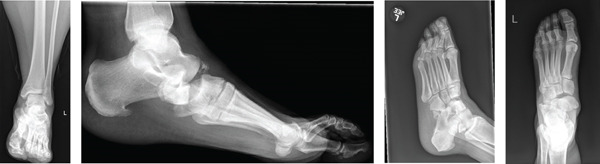
Anteroposterior and lateral injury radiographs.

**Figure 2:**
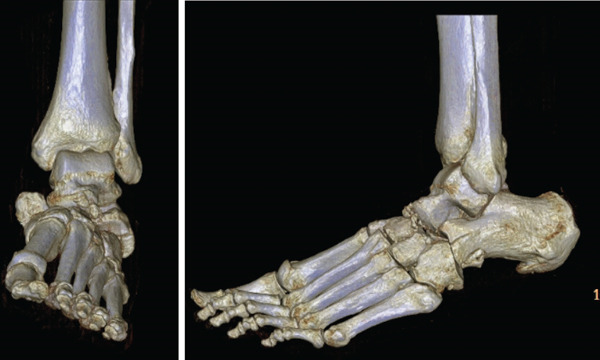
3D reconstruction CT scans of injured foot.

The patient is a 57‐year‐old female who presented to our emergency department (ED) complaining of left foot pain following a ground‐level fall from standing a few hours before arrival. The patient reported that she missed the final step while descending stairs, resulting in a twist of her foot and subsequent fall. Radiographs of the ankle and foot demonstrated a medial dislocation of the navicular with possible fracture of the cuboid and calcaneus (Figure 1). A computed tomography (CT) scan with 3D reconstruction was then obtained which demonstrated an inferomedial dislocation of the navicular without fracture, a fracture of the anterior/inferior calcaneus, a nondisplaced avulsion fracture of the cuboid, and small ossific fragments involving the lateral, middle, and medial cuneiform without frank midfoot instability otherwise (Figure 2). Orthopedic surgery was consulted secondary to her injury. On physical examination, there was an obvious deformity to the left foot with significant swelling and subcutaneous prominence noted over the medial aspect of her foot. Although there was skin tenting, the foot maintained adequate perfusion and remained neurovascularly intact. At this point, it was determined necessary to proceed with urgent transportation of the patient to the operating room, considering both closed reduction and open reduction as potential options, followed by surgical fixation of her injury.

**Figure 3:**
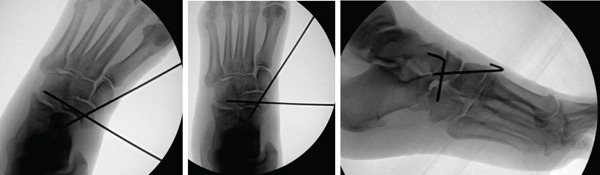
Intraoperative anteroposterior, oblique, and lateral views of reduction. Percutaneous pin stabilization of the navicular.

Following induction of general anesthesia and intubation by the anesthesiologist, the knee was flexed, and then distraction and abduction were applied through the forefoot. Gentle lateral pressure over the medial navicular was applied and a successful reduction of the navicular into the talonaviculocuneiform joint was obtained. Fluoroscopic images were taken to confirm adequate reduction. A 2.0‐mm Kirschner wire was inserted from medial to lateral traversing from the navicular to the cuboid. Subsequently, a second 2.0‐mm Kirschner wire was directed retrograde, originating from the medial cuneiform and entering into the navicular (Figure 3). The foot was gently manipulated and the navicular was found to be stable, supporting the lack of radiographic signs of gross midfoot instability. Consequently, we chose not to apply a spanning external fixator. Following the procedure, the patient′s foot was immobilized in a well‐padded splint, with instructions for nonweight bearing, and subsequently admitted to the hospital. A postoperative CT scan was performed to verify the adequacy of reduction and for operative planning for fixation of the anterior inferior calcaneus at the calcaneocuboid joint, aiming to restore the lateral column once swelling had improved. The patient remained hemodynamically stable postoperatively and was discharged with specific post‐op nonweight bearing instructions.

**Figure 4:**
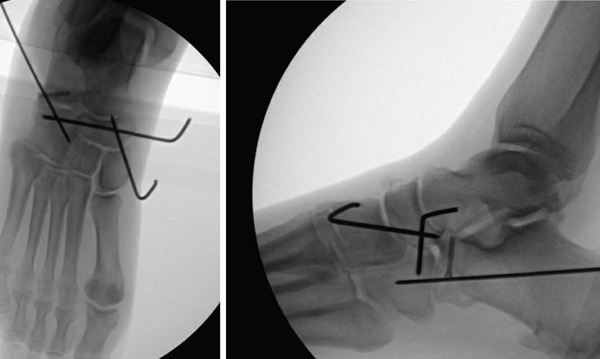
Intraoperative anteroposterior and lateral radiograph of anterior–inferior calcaneal process using Kirschner wire stabilization followed by 2.5‐mm compression screw.

**Figure 5:**
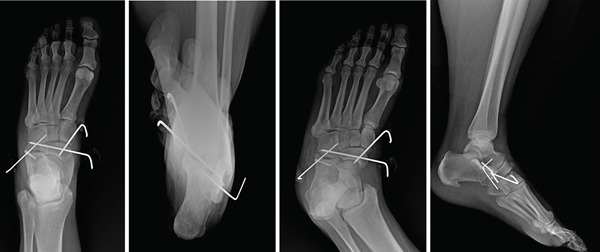
Anteroposterior, oblique, Harris view and lateral radiograph at 2 weeks post‐op.

During the patient′s initial postoperative visit to the orthopedic clinic, radiographs confirmed that the navicular and the entire midfoot were maintained in a reduced position. Her swelling had decreased and was deemed amenable to open reduction with fixation of her calcaneus fracture. Eleven days after the initial injury, the patient returned to the operating room and underwent open reduction internal fixation to address her anterior inferior calcaneus fracture. The fracture, which was displaced and split at the calcaneocuboid joint, was realigned and stabilized using a 2.5‐mm headless compression screw. Subluxation persisted at the calcaneocuboid joint, necessitating reduction with a 1.6‐mm Kirschner wire inserted across the joint while it was held reduced (Figure 4). She remained nonweight bearing in a posterior slab splint. The patient was closely monitored postoperatively. Ten days after surgery, the patient underwent evaluation in the orthopedic clinic, where x‐rays were obtained (Figure 5). She was progressing well postoperatively with no signs of infection, and her splint was replaced accordingly. The pins were removed at 8 weeks following the initial injury. At this stage, she remained nonweight bearing and was placed into a protective fracture boot. Physical therapy was prescribed to perform passive range of motion exercises for the foot and ankle.

**Figure 6:**
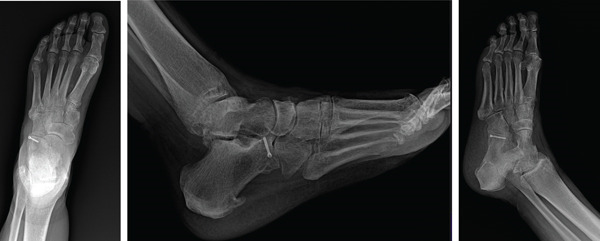
Anteroposterior, lateral, and oblique radiographs of left foot 8 months post‐op.

**Figure 7:**
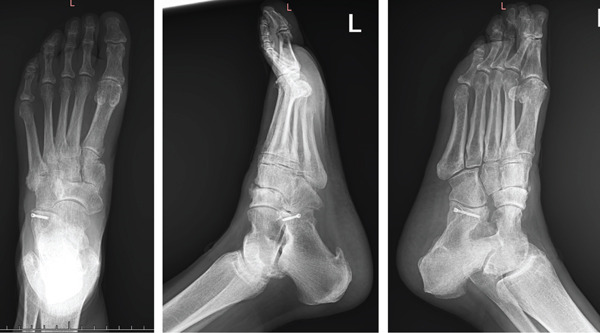
Anteroposterior, lateral, and oblique radiographs of left foot 15 months post‐op, note dorsal naviculocuneiform spurring.

At 12 weeks post‐op, the patient commenced active range of motion exercises with physical therapy and was permitted to bear weight as tolerated while wearing the fracture boot. X‐rays continued to demonstrate maintenance of talo–naviculo–cuneiform joint reduction. At 4 months postoperatively, the patient demonstrated limited active range of motion of the ankle with 65° of plantar flexion and 15° of dorsiflexion. She demonstrated the ability to perform a toe raise with her left foot and maintained neurovascular integrity. The transition to full weight bearing, without the fracture boot, was made at this time. At 8 months post‐op, the patient demonstrated a full active range of motion in plantar flexion and approximately 45° of dorsiflexion. Minor midfoot discomfort going up and down stairs was noted, but overall pain was well controlled with day‐to‐day activities. X‐rays at this time did not demonstrate any abnormalities (Figure 6). At 15 months post‐op, the patient had begun to notice increased feelings of instability of her ankle in the morning, with occasional dorsal foot swelling noted. Additionally, she reported experiencing pain correlating with increased activity levels, along with occasional numbness in the medial ankle/foot region. Radiographs taken during this visit revealed stable fixation without evidence of avascular necrosis (AVN) of the navicular. However, they did identify some dorsal calcifications over the naviculocuneiform joint (Figure 7). At this time, the recommended treatment included foot orthotics and activity modifications, with future monitoring advised for potential exostectomy if the dorsal exostosis becomes increasingly bothersome.

## 3. Discussion

The bony and surrounding ligamentous anatomy of the tarsal navicular is paramount to the structure and function of the foot. The navicular supports the medial column of the foot and functions as a keystone of the medial longitudinal arch [7, 8]. Isolated navicular dislocation is a misnomer. The literature demonstrates that navicular dislocations are associated with fractures of the navicular or other tarsals, midtarsal dislocations, and significant ligamentous injuries [3]. This is often the result of a high‐energy traumatic event. In the last decade, very few reports have identified complete navicular dislocations without associated navicular body fracture following a high‐energy trauma [2–5] and, to our knowledge, none after a low‐energy trauma. Multiple mechanisms have been proposed to describe the pathophysiology of navicular dislocations. Dhillon and Nagi describes the stability of the foot arising from the rigidity and ligamentous continuity of both the lateral and medial columns. For a complete dislocation of the navicular to occur without a fracture, disruption of the bony or ligamentous anatomy of the lateral column must also occur. A severe abduction/pronation force with the foot in plantar flexion can cause anatomic disruption to the medial and lateral columns. This can cause a transient midtarsal dislocation with subsequent relocation of the forefoot forcing the tarsal navicular to dislocate [1, 5, 6, 9]. Dixon suggests a transient midtarsal dislocation with a concomitant second direct blow causes dislocation of the navicular [13]. Others have suggested that forced plantar flexion with or without an axial load can lead to dislocation [6, 7, 14]. In reference to our injury, although CT scan did not demonstrate osseous signs of failure sites of the navicular, as discussed in Main and Jowett [14], there is needed disruption of dorsal and plantar ligaments to allow excursion of the navicular, this supports the analysis that there is inevitable damage to surrounding ligamentous support in these injuries [8]. The majority of the dislocations occur medially; however, the position of the foot and the direction of force determine the direction of dislocation [3]. Main and Jowett described a classification for injuries involving the midtarsal joints according to the direction of the deforming force. This includes the description of a “swivel” injury after the application of a medial or lateral force to the midfoot. A swivel dislocation after a medial force is applied to the forefoot may disrupt the talonavicular joint but leave the naviculocuneiform and calcaneocuboid joints intact. These injuries are often secondary to a fall from height [14].

Our case is unique in several facets, one in that is our patient suffered a ground‐level fall. Two, that aside from the obvious injury to the capsuloligamentous complex of the navicular, there was no gross ligamentous instability of the remaining tarsometatarsal complex demonstrated on radiographic imaging. This is with the caveat there was ossific injury to the calcaneus and cuboid demonstrating a complex midfoot/hindfoot injury constellation. Thirdly, the patient has no known prior history of connective tissue disorder, osteoporosis, chronic steroid use, or prior injury patterns that could indicate ligamentous laxity that could act as an explanation for this injury pattern. The position of the foot is unknown as the patient could not recall, but it could have been a pronation–abduction injury as she missed her step and the entire force of her body weight traveled through her foot, causing a midtarsal dislocation and disruption of the navicular. This could have caused the dislocation and the associated fractures of the cuboid and anterior inferior process of the calcaneus. Davis et al. described a similar fracture pattern in their case report in 2013 in a patient involved in a high‐speed motor vehicle accident. They believe the abduction force causes a strain at the bifurcate ligament which will result in avulsion fractures of the calcaneus and cuboid [3]. They liken this injury to perilunate dislocation of the wrist in which displacement of the lunate does not occur in isolation but depends on dislocation or relocation of the rest of the carpus [3, 12]. A contributing factor that may have predisposed this injury pattern is that although the mechanism is “low energy”, our patient did notably fall into Class II obesity with a BMI of 35 at the time of injury which may have played a role in converting this to a pseudo‐higher energy injury.

Multiple treatment options have been described in the literature, mostly from isolated case reports. Treatment is often guided by the extent of the associated bony and ligamentous injury as well as the postreduction stability of the midfoot joints. Secondary to the mechanisms described above, the ligamentous disruption often lends surgical fixation due to disruption of the medial and lateral columns. However, nonoperative treatment has been described [12, 15]. The goal of operative intervention is to achieve prompt and stable anatomical reduction [2]. Operative techniques described include closed reduction with percutaneous pinning [3, 5, 7, 9], open reduction and pinning [4, 6, 12], primary arthrodesis [2, 7], and adjunctive use of external fixation [3]. In our case, we were able to utilize CRPP to obtain anatomic stable reduction of the navicular. Of the four cases of total navicular dislocation described in the last decade, similar post‐op protocols were followed. In three cases, pins were removed at 6 weeks, and the patients were instructed to remain nonweight bearing for 3 months. Weight bearing was then gradually progressed at that time [2, 4, 5]. In the fourth case, pins and external fixation were removed at 7 weeks postoperatively. However, the patient was lost to follow‐up [3]. No significant complications were noted in these reports. However, follow‐up was short.

Secondary to the scarcity of the literature, little is known about the complications after total navicular dislocation. The blood supply to the navicular is primarily via small branches of the dorsalis pedis and tibialis posterior arteries from the medial pole and the dorsal and plantar surfaces with a relatively avascular central third [8]. Often after dislocation, the attachment of the tibialis posterior may be the only soft‐tissue structure remaining and become vital to its remaining blood supply [12]. AVN has been reported after total dislocation [6, 12, 16]. Other complications have been described including residual subluxation of the navicular, flatfoot deformity leading to midfoot collapse, posttraumatic arthritis, and stiffness [3, 6, 9, 12, 17].

Complete dislocations of the tarsal navicular, without fracture of the navicular, are rarely mentioned in orthopedic literature, with only 15 reported cases since 1924 [3]. After a thorough review of the literature, we did not find a single case where isolated navicular dislocation was caused by low‐energy trauma. Jung et al. described a case where a 78‐year‐old female suffered an inversion injury after falling downstairs which resulted in an isolated medial talonavicular dislocation [11]. Although extremely rare, this does not describe our injury with a complete isolated dislocation of the tarsal navicular. Midtarsal dislocations in general are associated with higher energy injuries as it takes a considerable amount of force to disrupt the strong ligamentous structure of the midfoot. Although patients′ obesity status may have contributed to greater force concentrated through the injury site, this alone is insufficient to fully explain this injury pattern. In conclusion, isolated navicular dislocation without fracture following a low‐energy injury is poorly described in the orthopedic literature, and we believe our case report not only adds to a limited amount of information on the management of isolated navicular dislocation but also provides valuable 15‐month follow‐up data that demonstrates favorable functional recovery with also apparent risk of long term sequelae of traumatic injury which encourages that these patients should be followed long term.

## Funding

No funding was received for this manuscript.

## Consent

The authors have nothing to report.

## Conflicts of Interest

The authors declare no conflicts of interest.

## Data Availability

Data sharing is not applicable to this article as no datasets were generated or analyzed during the current study.
